# An integrated model for interdisciplinary graduate education: Computation and mathematics for biological networks

**DOI:** 10.1371/journal.pone.0257872

**Published:** 2021-09-28

**Authors:** Kelsey E. McKee, Daniel Serrano, Michelle Girvan, Gili Marbach-Ad

**Affiliations:** 1 College of Computer, Mathematical and Natural Sciences, Teaching and Learning Center, University of Maryland, College Park, Maryland, United States of America; 2 Institute for Research in Electronics and Applied Physics, University of Maryland, College Park, Maryland, United States of America; 3 Department of Physics, University of Maryland, College Park, Maryland, United States of America; Al Mansour University College-Baghdad, IRAQ

## Abstract

The current challenges at the forefront of data-enabled science and engineering require interdisciplinary solutions. Yet most traditional doctoral programs are not structured to support successful interdisciplinary research. Here we describe the design of and students’ experiences in the COMBINE (Computation and Mathematics for Biological Networks) interdisciplinary graduate program at the University of Maryland. COMBINE focuses on the development and application of network science methods to biological systems for students from three primary domains: life sciences, computational/engineering sciences, and mathematical/physical sciences. The program integrates three established models (T-shaped, pi-shaped and shield-shaped) for interdisciplinary training. The program components largely fall into three categories: (1) core coursework that provides content expertise, communication, and technical skills, (2) discipline-bridging elective courses in the two COMBINE domains that complement the student’s home domain, (3) broadening activities such as workshops, symposiums, and formal peer-mentoring groups. Beyond these components, the program builds community through both formal and informal networking and social events. In addition to the interactions with other program participants, students engage with faculty in several ways beyond the conventional adviser framework, such as the requirement to select a second out-of-field advisor, listening to guest speakers, and networking with faculty through workshops. We collected data through post-program surveys, interviews and focus groups with students, alumni and faculty advisors. Overall, COMBINE students and alumni reported feeling that the program components supported their growth in the three program objectives of Network Science & Interdisciplinarity, Communication, and Career Preparation, but also recommended ways to improve the program. The value of the program can be seen not only through the student reports, but also through the students’ research products in network science which include multiple publications and presentations. We believe that COMBINE offers an effective model for integrated interdisciplinary training that can be readily applied in other fields.

## Introduction

Scientists in today’s workforce frequently face problems whose solutions require a diverse set of methods and ideas [1–4]. This is the hallmark of interdisciplinary problem solving and is especially true in network science, which has emerged as a new collaborative field [5, 6]. Network science focuses on how the interaction patterns among a system’s constituent components shape its overall function. Network science has been widely applied to address physical, biological, technological, and social problems in which complex interaction patterns play an important role. Researchers working on these types of issues, need expertise in related fields as well as the ability to communicate and collaborate with stakeholders outside of their academic disciplines [7, 8]. As a result, there is a growing consensus [9–12] that developing an interdisciplinary skill set should be part of the graduate training process.

To understand the breadth of expertise needed to tackle problems that span multiple disciplines, consider the example of the rapid research effort that emerged in the wake of the COVID-19 pandemic. Hypothetically, a graduate student in epidemiology exploring the transmission of infectious disease may want to use network science to explore how social interaction patterns impact transmission [13]. To this end, the student may decide to independently learn and apply computational network models to their own research. Alternatively, they may collaborate with an applied mathematics student to jointly create a model of transmission. To forge a successful collaboration, these students must communicate their research in a way the other can understand, and think creatively about how to integrate theory and methods from each other’s fields [14, 15].

Here we describe the design of and students’ experiences in the COMBINE (Computation and Mathematics for Biological Networks) interdisciplinary graduate program. COMBINE was created to provide high-quality training in the development and application of network science to biological systems for students drawn from three primary domains: life sciences, computational/engineering sciences, and mathematical/physical sciences. COMBINE integrates expertise and methods from these three domains to gain insights into biological networks across scales: from biomolecular networks to neuronal networks to ecological networks. The COMBINE program is supported by the National Science Foundation’s Graduate Research Training grant (NRT) and uses an integrated model of interdisciplinary graduate student training in which students gain knowledge and experience in network science as a supplement to their own doctoral program.

One goal of this paper is to illustrate the process of designing and implementing a graduate training program that aligns program objectives, program components, and educational training models. In addition, we describe students’ experiences in the program, as well as insights from their advisors, to illustrate the strengths and challenges associated with this type of interdisciplinary graduate training program. We hope that this paper can serve as a road map for other training programs that wish to provide integrated interdisciplinary education and that the lessons learned in the COMBINE program can advance the efforts of future educational programs.

### Interdisciplinary research and graduate training

**Fig 1:**
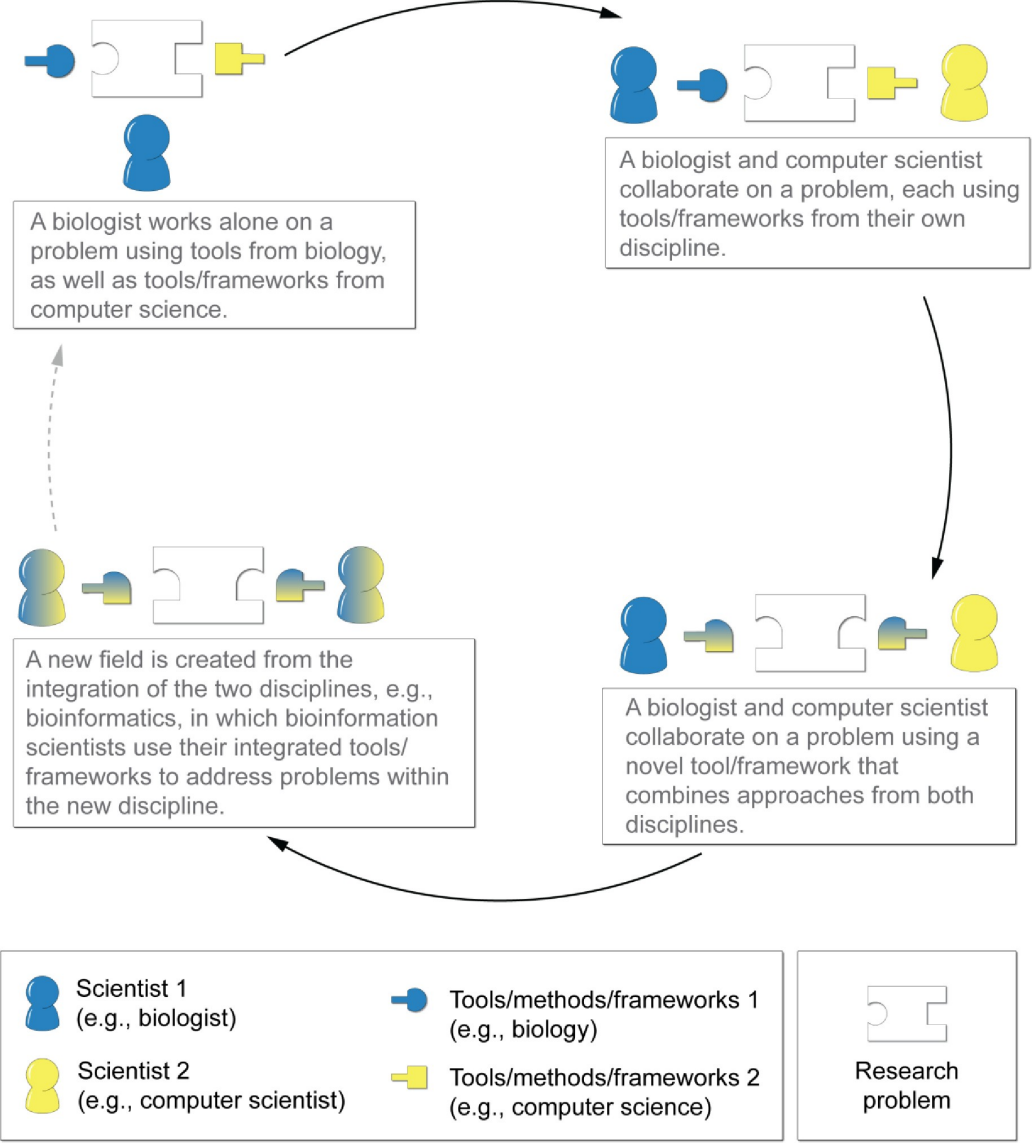
The spectrum of interdisciplinarity. *Note*. The dashed arrow represents the cycle beginning again. For example, with the field of bioinformatics, a bioinformaticist incorporates tools/frameworks from another outside discipline.

There are many definitions for interdisciplinary research. Klein [16] characterizes interdisciplinary research as a spectrum (Fig 1) where interdisciplinary scientific study is categorized by the degree of integration of methods and collaborators from different fields, from intradisciplinary to crossdisciplinary to transdisciplinary work. At one end of the integration spectrum, a single person incorporates knowledge, data, and methods from different disciplines to address a problem. From there, increased integration is two or more researchers from different disciplines collaborating on a research problem, each using the expertise from their own discipline and combining results into joint findings. Even more integration happens when two or more researchers from different disciplines collaborate on research using a *shared framework* to identify connections and interactions between the knowledge, data, and methods of their disciplines. Finally, full integration is when a group of researchers deeply integrate multiple disciplines to create novel frameworks, new paradigms, or new disciplines. The ideal level of integration is unique to each problem and research setting. Here we use the term interdisciplinary to refer to the entirety of the spectrum.

**Fig 2:**
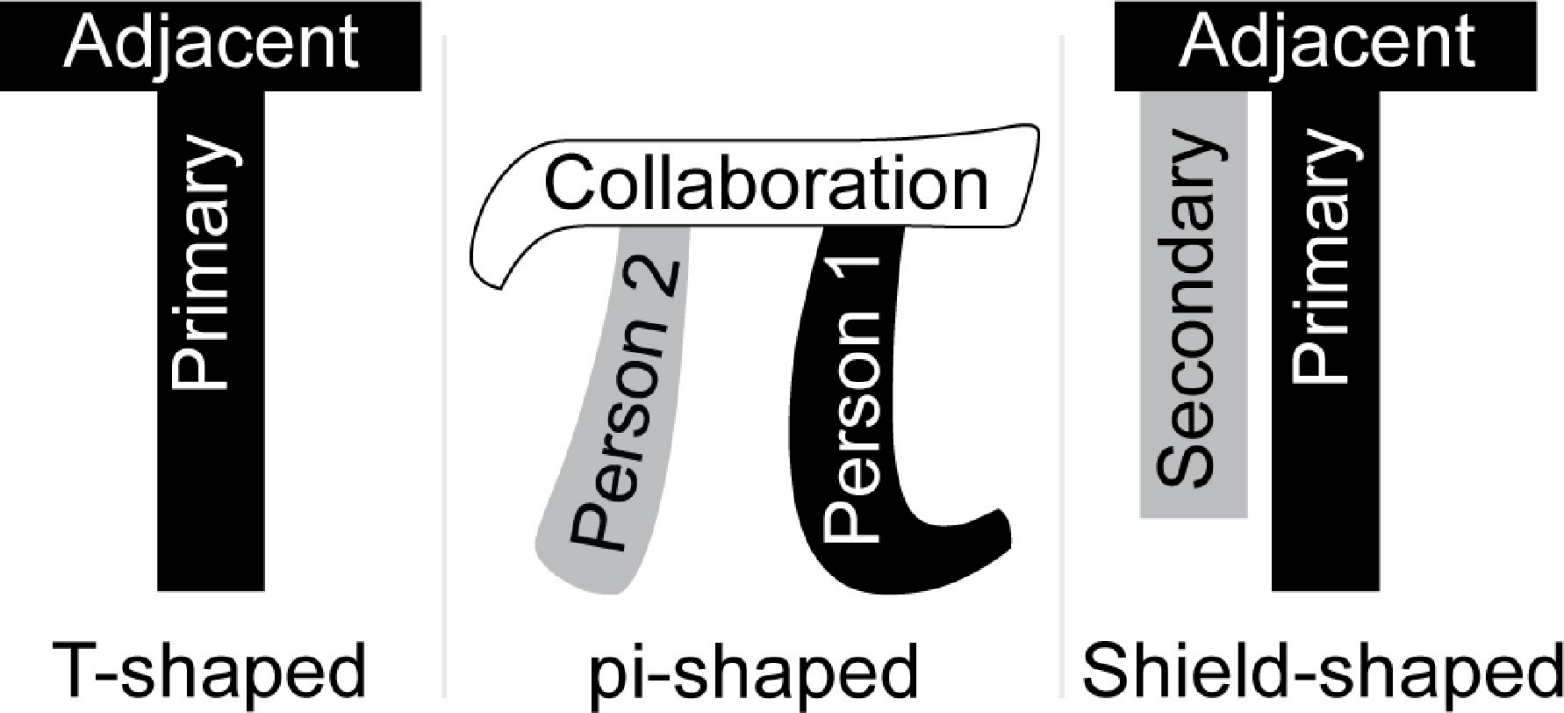
Models of interdisciplinary graduate education adapted from Pennington et al. [19]. *Note*. Adjacent, primary, and secondary refer to disciplines (e.g., primary discipline).

Recommendations and guidance on how to train graduate students to become interdisciplinary scholars can be found in the literature [9, 10, 17, 18]. Pennington and colleagues [19] review and discuss three different commonly used models of interdisciplinary education (Fig 2): the T-shaped model [20–23], the pi-shaped model [24], and the shield-shaped model [10]. In the T-shaped model graduate students receive in-depth expertise within a specific discipline (i.e., the vertical leg of the T) along with basic knowledge of similar fields (i.e., the breadth at the top of the T). The pi-shaped model usually refers to bringing experts in different fields together (i.e., one expert in each leg) in order to collaborate (i.e., the top of the pi). The shield-shaped model expands students’ expertise in a secondary discipline so they can integrate that knowledge and apply it to their own work, as well as to facilitate collaboration with others through their shared expertise. The shield-shaped model differs from the pi-shaped model because it assumes that the additional expertise in a secondary discipline is sufficient for collaboration, while the pi-shaped model focuses explicitly on training in collaboration and communication.

Each of these approaches prepares students to work at a specific level of the spectrum of interdisciplinary integration. For example, the shield-shaped model prepares students to work at lower levels of integration, as it focuses on interdisciplinary application within an individual’s own work but not on collaboration skills (e.g., a biologist learning computational modeling and applying it to their own work in biology). Meanwhile, the pi-shaped model prepares students to collaborate across fields but does not focus on expanding the diversity of expertise within an individual student. Yet, the ideal level of integration is unique to each problem and research setting [25]. As such, graduate students must be prepared to work at any level of interdisciplinary integration to be ready to address modern problems.

**Fig 3:**
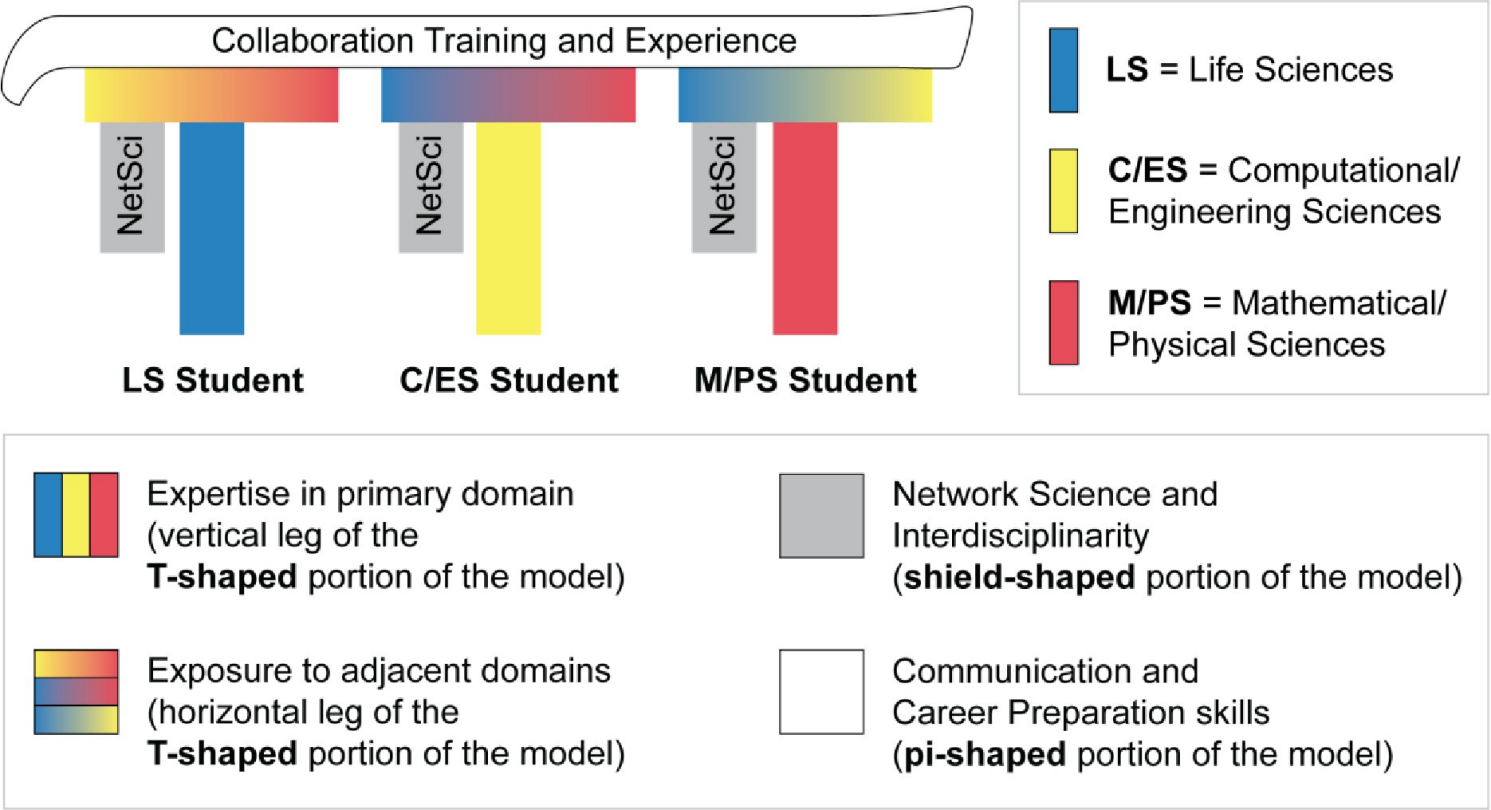
The COMBINE model of integrated interdisciplinary graduate education.

COMBINE utilizes a combination of these three models to prepare students to be well-rounded interdisciplinary scholars in network science (Fig 3). COMBINE builds upon a student’s expertise that is developed in their home program (i.e., the vertical leg of the T-shaped model) by creating moderate expertise in domains relevant to network science through discipline-bridging coursework outside the student’s primary domain (i.e., the horizonal portion of the T-shaped model), as well as specific training in network science as a secondary discipline (i.e., the shield-shaped model). Finally, the program provides training in collaboration between students from different disciplines (i.e., pi-shaped model). As such, COMBINE was designed not only to develop graduate students with exposure to other domains and competency in network science, but also promote their ability to collaborate.

It is particularly important that COMBINE operates at the intersection of the three models in order to train strong network scientists. A primary feature of the field of network science is the collaboration between a wide variety of disciplines, including computational science, mathematics, physics, quantitative biology, and the social sciences [5, 6, 26]. The goal of this emerging field is to use networks to represent, analyze, model, and predict interaction patterns in order to gain insight into the behavior of complex systems. While network science has made significant strides in bringing together researchers from across disciplines based on common questions, it still faces the cultural and communication barriers seen in other interdisciplinary contexts [19]. COMBINE was designed to dissolve these barriers by providing training in network science to students from different disciplines side-by-side so that, in helping to educate each other, they may discover the convergence of their differing perspectives and pave the way for ground-breaking new research.

### Engaged graduate education programs

The higher education literature provides insight into how interdisciplinary education can be done in a high-quality and sustainable way, regardless of the content area [27–30]. Haworth’s and Conrad’s [31] engagement theory identifies four aspects of a successful interdisciplinary graduate education program. First, the program should recruit participants from diverse backgrounds (e.g., disciplines, gender, ethnicity). Research illustrates the strengths of programs that include diverse members [1, 32], stressing that “effective decisions regarding complex, multifaceted problems require the consideration of multiple perspectives” ([33], p. 1107). Second, the program should foster a participatory culture. Newswander and Borrego [28] assert that students and faculty should be partners in the academic process. They suggest a participatory culture should be promoted through classes, lectures, workshops, and other informal activities. Participatory culture is known to enhance collaboration, greater professional preparation, as well as problem-solving and leadership skills [31]. Third, the program should provide wide opportunity for interactions in and outside the classroom, encouraging peer learning, and leadership and mentorship roles [34, 35]. These opportunities could include engaging students in research projects, peer review of projects, and creating shared products (e.g., tutorials). Finally, if graduate students are expected to expand upon their traditional graduate program they must be provided with adequate resources, such as financial, administrative and emotional supports [28, 31].

All of these elements were incorporated into the design of COMBINE. The program aims to intentionally recruit a diverse set of students from disciplines related to network science. Program activities, described in detail below, provide a variety of formal and informal educational experiences to students to allow for students’ leadership and contributions. Lastly, the financial structure of the program provides robust support for administration as well as to the students directly. As such, tenets of engagement theory are woven throughout COMBINE.

### The COMBINE program

**Fig 4:**
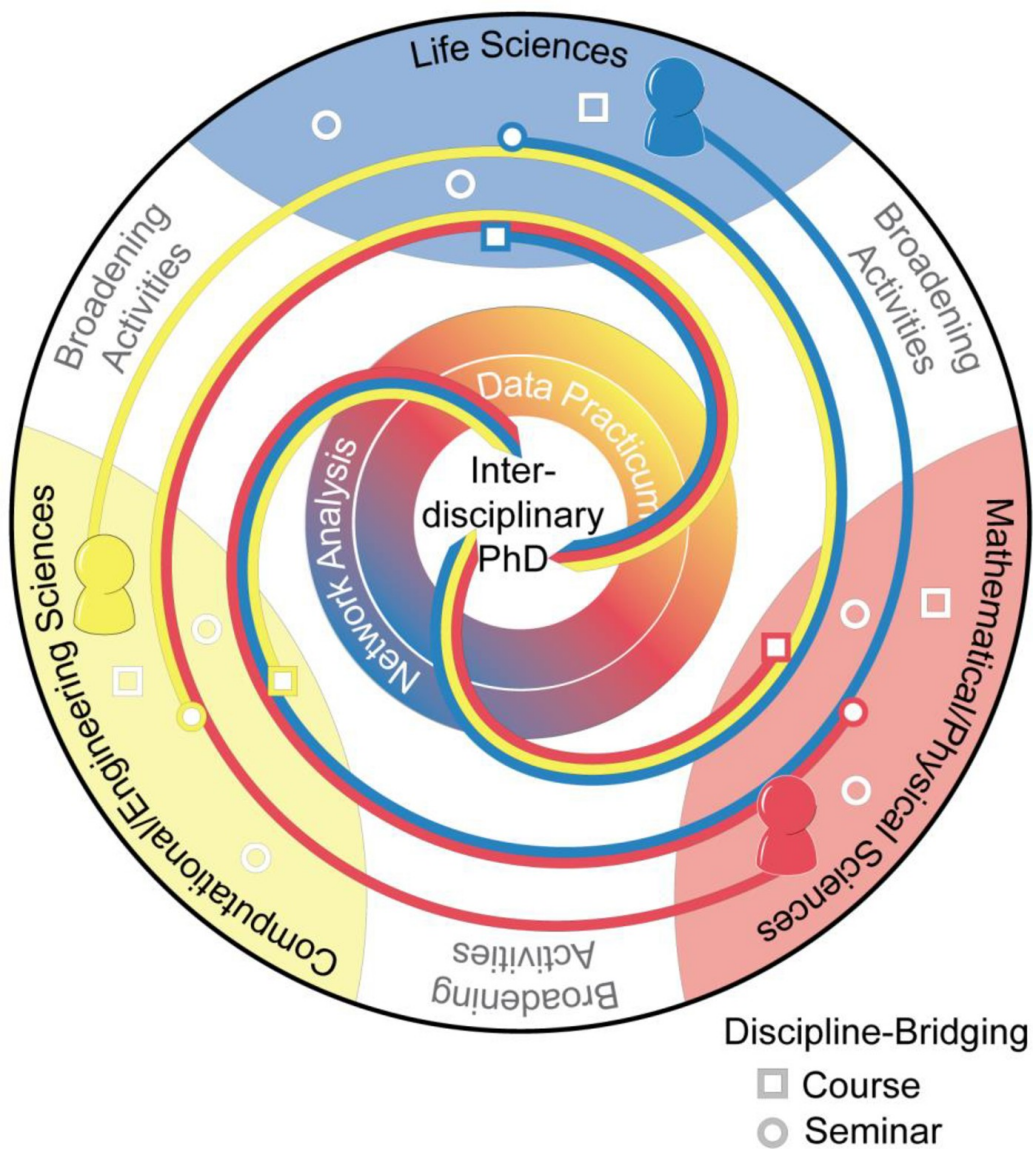
The COMBINE program.

COMBINE was designed to provide students with skills necessary to work in the interdisciplinary field of network science, with a focus on biological applications, through a constellation of experiences that promote a high level of engagement from students and faculty and will ideally be established as a permanent feature of graduate education at the University of Maryland, College Park (Fig 4). The program is composed of students and faculty working in one of three discipline domains: Life Sciences (LS), Computational/Engineering Sciences (C/ES), and Mathematical/Physical Sciences (M/PS). These three domains each contribute important elements towards research efforts in network science, especially within the area of network biology. The C/ES domain provides computational frameworks and tools needed for the analysis of complex network data. The LS domain provides critical application area expertise, such as mechanistic and systematic understandings of biological phenomena, that ground and establish constraints on the C/ES tools and M/PS theories. Finally, the M/PS domain provides theoretical and modeling frameworks that can extrapolate beyond the scope of LS studies, allowing for interrogation of the complex dynamics across biological scales.

### COMBINE objectives and components

The skills needed to conduct integrated, interdisciplinary research applying network science to biological systems are articulated in the 12 program objectives (Table 1). These were designed to supplement the skills gained from a student’s home program, where they receive in-depth, specialized training in their primary discipline. The COMBINE objectives are grouped under three broad categories: Network Science and Interdisciplinarity, Communication, and Career Preparation. Together, the objectives promote students’ expertise in network science (shield-shaped portion of the model), familiarity with related fields (T-shaped portion of the model) as well as their collaboration skills (pi-shaped portion of the model, which includes communication and career preparation skills).

**Table 1:** COMBINE program objectives.

Category	Objective
Network Science & Interdisciplinarity	(1) Learn about important research results in network science
	(2) Learn about the theory and methods of network analysis
	(3) Develop an understanding of methods of other fields
	(4) Apply methods of network analysis to biological data
Communication	(5) Develop skills for oral presentations
	(6) Learn to build effective visualizations
	(7) Develop written communication skills
	(8) Communicate to a diverse audience
Career Preparation	(9) Build teamwork skills
	(10) Gain experience mentoring others
	(11) Develop leadership skills
	(12) Gain exposure to diverse career options

*Note*. Though all of the objectives listed under the “Communication” category can be considered Career Preparation, communication skills were a specific target of the COMBINE program and thus separated into their own category. The Communication and Career Preparation categories together make up the objectives that target collaboration.

To reach these objectives, the basic structure of COMBINE is that doctoral students simultaneously complete their home PhD program requirements and the COMBINE program components (Table 2). Since COMBINE recruits students from diverse disciplinary backgrounds, the overarching goal is to provide a program with some core mandatory components, but also to allow student flexibility with options for fulfilling program requirements.

**Table 2:** COMBINE program components.

Component	Description
***Core Interdisciplinary Courses & Seminars (required)***	
Computational and Mathematical Analysis for Networks Across Scales (*Network Analysis*)	A 3-credit course where students, working in small groups to complete assignments, learn to apply network science methodologies to large datasets drawn from life science applications. Modules include four themes: network measures, mechanistic network models, network statistics and machine learning, and network visualization.
Network Science Literature Survey	A 1-credit seminar in reading group format in which students discuss seminal network biology papers.
Network Biology Research-in-Progress	A 1-credit seminar in which each student presents their own research and obtains peer and instructor feedback. Both seminars feature ~3 invited faculty presentations, and opportunities for students to practice presentations.
Interdisciplinary Research & Communication Practicum for Data-Driven Science (*Data Practicum*)	A 3-credit course where students learn to communicate their research to interdisciplinary audiences through oral and written presentations and peer evaluation. Students work on an independent research project in network science outside of class that they use in the communication pieces.
***Discipline-Bridging Courses & Seminars (required*, *content specific to each student)***	
3–4 Credit Elective Course	A 3-credit course from one of the 2 domains outside the student’s home domain, chosen from a long list of options.
1 Credit Elective Seminar	A 1-credit seminar from the student’s other non-home domain. The purpose of the discipline-bridging courses is for students to gain familiarity in areas outside their home domain that are relevant to their research.
***Broadening Activities (required*, *activity and/ or role specific to each student)***	
Peer-to-Peer Tutorials (P2P)	Groups of 3–5 students work to prepare a tutorial on a topic of their choosing with guidance from COMBINE faculty. The tutorial is delivered to peers both within and outside the COMBINE program.
COMBINE Annual Symposium	A symposium that showcases COMBINE research activities with presentations by COMBINE students, presentations from leading researchers, short talks from COMBINE faculty, and panel discussions.
Career Development Workshop	Annual workshop on how network-focused data science methods can be applied in a variety of career contexts. The workshop is largely student-organized, with COMBINE faculty oversight.
Outreach	Outreach activities that promote interdisciplinary STEM education. Activities include outreach to middle/high school students and the general public and undergraduate research mentoring.
Peer Mentoring	3-person student teams from across domains and graduate program stages. Peer mentoring teams meet 2–3 times per year to provide peer support and discuss progress towards research and career goals.
***Additional Features***	
Lightning Workshops	Annual event in which faculty and students from the COMBINE program and the broader network science community deliver brief presentations on their work and brainstorm about interdisciplinary projects.
Out-of-Field Mentors	A faculty member from outside the student’s domain to provide advice about the student’s path through the program, coursework, and research. Out-of-field mentors usually serve on students’ dissertation committees.
Fellow Meetings	Students meet 2–3 times per semester over coffee for guided activities designed to develop a sense of community.
COMBINE Student Committee	A group of students elected to generate and implement initiatives that enhance the program. The Committee is divided into roles: Chair, Undergraduate Research, Program Presence, Outreach, Professional Development.
Internships with COMBINE partners	Optional internships with industry and government partners, who are invested in the translation of scientific advancements to a range of applications, are encouraged to help students explore various potential career paths.

The program components (Table 2) largely fall into three categories: (1) core coursework that provides content expertise, communication, and technical skills, (2) discipline-bridging elective courses in the two COMBINE domains that complement the student’s home domain, (3) broadening activities such as workshops, symposiums, and formal peer-mentoring groups.

Beyond these components, the program builds community through both formal and informal networking and social events. In addition to the interactions with other program participants, students engage with faculty in several ways beyond the conventional advisor framework, such as the requirement to select a second out-of-field advisor, listening to guest speakers, and networking with faculty through workshops.

Program components were designed to address objectives either *directly* (D) as a primary function of the component, *indirectly* (I) as natural consequence of the efforts to support primary functions, or *not at all* (N; Table 3). Consider the two core courses as an example. The network analysis course is intended to directly address the Network Science & Interdisciplinarity objectives “*learn about important results in network science*” and “*learn about the theory and methods of network science*” through course readings and lectures, as well as “*apply methods of network analysis to biological data*” through course assignments. The Communication and Career Preparation objectives are not directly taught in the course, but are rather indirectly targeted through presentations, written assignments, and group projects. Meanwhile, in the data practicum course, Communication objectives are directly targeted through instruction on how to create an effective visualization, while the network science objectives are indirectly targeted through students’ exposure to network science by observing their peers’ research presentations [36].

**Table 3:** Planned alignment between COMBINE objectives and components determined by the leadership team.

Objective	Core Courses		COMBINE Seminars		Discipline-Bridging Courses		Broadening Activities				
	Data Practicum	Network Analysis	Literature Survey	Research-in-Progress	3-4-Credit Elective	Elective Seminar	Peer-to-Peer Tutorial (P2P)	COMBINE Annual Symposium	Career Development Workshop	Outreach	Peer Mentoring
***Network Science & Interdisciplinarity***											
Learn about important research results in network science	I	D	D	I	N*	N*	N	D	N	N	N*
Learn about the theory and methods of network analysis	I	D	D	I	N*	N*	N	I	N	N	N*
Develop an understanding of methods of other fields	D	D	D	D	D	D	I	I	N*	N	N*
Apply methods of network analysis to biological data	D	D	N	I	N*	N*	N	N	N	N	N
***Communication***											
Develop skills for oral presentations	D	I	D	D	N*	N*	D	I	N	N*	N
Learn to build effective visualizations	D	D	N	I	N*	N*	I	I	N	N*	N
Develop written communication skills	D	I	I	N	N*	N*	N	N	N	N*	N
Communicate to a diverse audience	D	I	I	D	I*	I*	D	I	N	D	N
***Career Preparation***											
Build teamwork skills	N	D	D	N	N*	N*	D	N	I	N*	I
Gain experience mentoring others	N	N	N	N	N	N	N	N	N	N*	D
Develop leadership skills	N	I	N	N	N	N	D	N	I	N*	N
Gain exposure to diverse career options	N	N	I	I	N	N	N	I	D	N*	N

*Note*. D = Directly targeted (dark gray); I = Indirectly targeted (light gray); N = Not targeted (white).

* indicates that the degree to which the component was expected to target the objective varied based on either the specific course/activity or specific role selected by each student.

The program allows students to customize their own experiences to a certain degree. This flexibility includes the courses students select as electives, the specific activities in which they participate to satisfy COMBINE requirements (e.g., satisfying the outreach requirement by mentoring undergrads or presenting the COMBINE program to an outside group), the projects they complete as part of their coursework, their role in different activities (e.g., an event facilitator or an attendee), and the degree to which students expand upon the basic structure of the program (e.g., connecting with a guest speaker for career advice). This flexibility allows students to tailor the program to their specific needs, but also leads to variability in the degree to which the different program components contribute to gains in the program objectives for individual students.

#### Program administration

The original design for the structure of the program consisted of five principal investigators, faculty members providing direct support to the program through teaching core courses or participating in other program components, various other faculty working in fields related to network science who advise student participants and/or participate in program components, two program administrators who run the logistical and organizational aspects of the program, internal and external evaluation teams, an expert advisory board, and five cohorts of graduate student fellows and their advisors.

The COMBINE internal evaluation program utilizes a mixed-method, multi-informant evaluation that includes surveys, individual interviews, observations and focus groups. The internal evaluation team consists of an expert evaluator (i.e., a research professor in science education) and a graduate assistant. The evaluation team has been involved in the program from its creation and is acknowledged as an outside entity that provides ongoing feedback to the program leadership team and advisory board.

## Methods

### Participants

Students and faculty from diverse disciplines were intentionally recruited to participate in the COMBINE program and selected as students from a competitive pool of applicants. Seventy students make up the five cohorts of COMBINE students (Tables 4 and 5). The distributions of gender and ethnicity are generally representative of the current distributions in doctoral programs for the disciplines involved [37].

**Table 4:** COMBINE student demographics upon starting the program.

	Percent of COMBINE Students	Percent of Analytic Sample
	(*N* = 70)	(*N* = 24)
*Doctoral Program Coursework Stage*		
1 year or less	49%	29%
2 years	34%	42%
3 years	11%	17%
4 or more years	6%	13%
*Gender*		
Male	64%	67%
Female	36%	33%
*Race/ Ethnicity*		
White	46%	65%
Black or African American	3%	4%
Asian or Asian American	27%	15%
Hispanic or Latino	4%	8%
Other	21%	8%
*Career Goals*		
Academia	33%	40%
Researcher	19%	25%
Industry	24%	23%
Unknown	10%	8%

*Note*. Race/Ethnicity and Career Goals reflect the percentage of students who endorsed each category. Students who endorsed multiple categories are counted in each category, though all responses are weighted so the total reflects the total number of students in the program. Analytic sample includes students from the first two cohorts, for which post-program data are currently available.

**Table 5:** COMBINE student and faculty disciplines.

Domain	Percent of Students		Percent of Faculty	
	All	Analytic Sample (*N* = 24)	All	Analytic Sample (*N* = 14)
	(*N* = 70)		(*N* = 45)	
Computational/Engineering Sciences	29%	33%	29%	21%
Life Sciences	40%	33%	35%	50%
Mathematical/Physical Sciences	31%	33%	27%	29%
Other	--	--	9%	--

*Note*. Student analytic sample includes students from the first two cohorts, for which post-program data are currently available. Faculty included here are those involved in the program as advisors, affiliates, instructors, and/or program administrators. The faculty included in the analytic sample are the advisors of students from the first two cohorts for which post-program data are available (i.e., those who completed the post-program advisor survey).

As COMBINE is supported by an NRT grant, competitive students that are eligible (i.e., are U.S. citizens) receive financial support in the form of a one-year stipend (around half of the students). Students who do not receive the stipend can still benefit from other funding support, such as travel for conferences. Upon completion of the program, all students are eligible to obtain an official COMBINE graduate certificate from the university.

### Data collection and analyses

Here, we focus our attention on the first two cohorts of students (*N* = 24), for whom post-program data are currently available. Tables 4 and 5 show that the students included in the analytic sample are largely representative of the students in all five cohorts with two exceptions: (1) the analytic sample has a larger proportion of White students than the fully sample and (2) the analytic sample has more 2^nd^ year and fewer 1^st^ year students than the full sample.

To describe students’ experiences of the COMBINE model, we draw upon data from the COMBINE evaluation program. The data presented here were collected by the evaluation team [38]. Informed consent (written for surveys and oral for interviews) included a disclaimer that only the evaluation team would have access to identifiable data, and the leadership team would have access to aggregated, de-identified data. The study includes human participants and was approved by University of Maryland IRB. The number and title of the IRB: [927445] NRT-DESE: Network Biology: From Data to Information to Insights.

Adapted versions of validated post surveys [39] were used to collect end-of-program data from both the students and from their faculty advisors. The students’ post-program survey was administered immediately after their completion of the two year (Cohort 1) or two and a half year (Cohort 2) program. Advisors for students in both cohorts were surveyed in the summer of 2020 which was one year after program completion for Cohort 1 and six months after program completion for Cohort 2. Face validity of the adapted surveys was established by the evaluation and program leadership teams. The surveys were administered online via Qualtrics and included questions about the student’s experience in the program or the advisor’s impressions of their student’s experience in the program, using a variety of question formats including scale-response and open-ended questions. One student in the first cohort did not respond to requests to complete the post-program survey and was thus omitted from the quantitative analyses. Individual interviews (*N* = 8 students) and focus groups with all students were conducted following their completion of the program and were audio recorded and transcribed for analysis. Two of the faculty advisors included in the analytic sample (*N* = 14) each advised two students in the analytic sample. They were asked to complete the same post program survey twice, once for each student. Thus the functional sample size for the faculty advisor survey is 16. The faculty advisors for seven students in the analytic sample did not respond to requests to complete the post program survey, and are therefore missing from the analysis. Additionally, one survey was omitted in order to ensure unbiased results as it was completed by an advisor who is a member of the COMBINE leadership team and an author of this paper (MG), for a total of eight missing advisor surveys.

**Fig 5:**
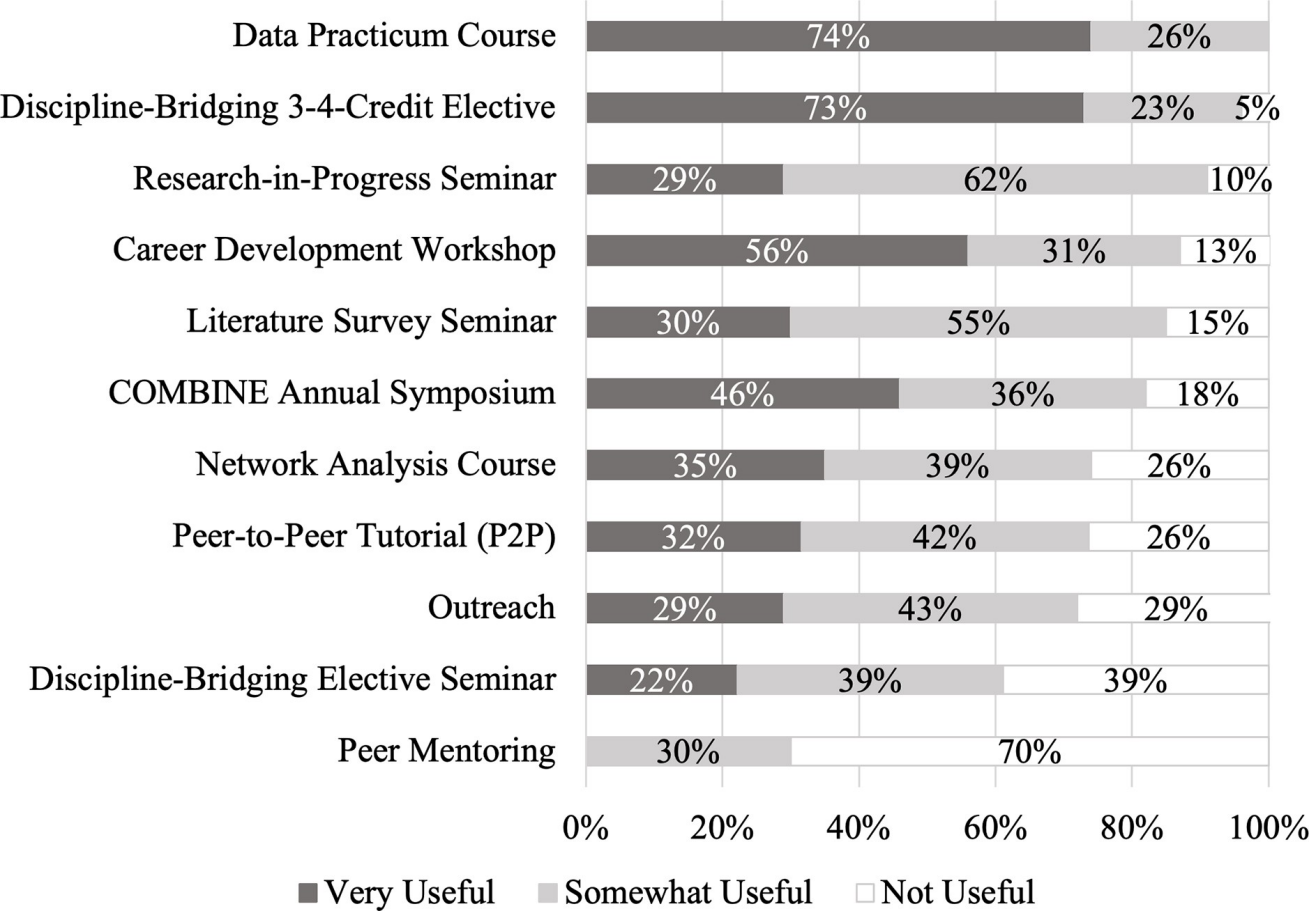
Student ratings of the usefulness of COMBINE components. *Note*. Percentages were calculated out of the total number of students who participated in each component: Data Practicum Course (*n* = 23), Discipline Bridging 3-4-Credit Elective (*n* = 22), Research-in-Progress Seminar (*n* = 21), Career Development Workshop (*n* = 16), Literature Survey Seminar (*n* = 20), COMBINE Annual Symposium (*n* = 22), Network Analysis Course (*n* = 23), Peer-to-Peer Tutorial (P2P; *n* = 19), Outreach (*n* = 21), Discipline-Bridging Elective Seminar (*n* = 18). Only students in the second cohort were asked about the usefulness of Peer Mentoring (*n* = 10). Percentages may sum to greater than 100% due to rounding.

The quantitative illustrations of students’ descriptions of their experience in the COMBINE program were drawn from the survey. Students were provided with a list of all program components (Table 2) and objectives (Table 1) and asked, “For the COMBINE components that you participated in, check those that helped you with each of the following objectives.” We calculated the percentage of students that reported that a given program objective was met by a given component (out of the total number of students who participated in each component; Table 6). For each program objective, we also calculated the percentage of students who reported that it was met by at least one component (Table 6, right-most column). Using data from a different question in the post-program survey, we calculated for each program component the percentage of students who reported it as ‘very useful’, ‘somewhat useful’, or ‘not useful’ (out of the total number of students who participated in that component; Fig 5).

**Table 6:** Student reports of whether COMBINE objectives were met by each component.

Objective	Core Courses		COMBINE Seminars		Discipline-Bridging Courses		Broadening Activities					Addressed by any component
	Data Practicum	Network Analysis	Literature Survey	Research-in-Progress	3-4-Credit Elective	Elective Seminar	Peer-to-Peer Tutorial (P2P)	COMBINE Annual Symposium	Career Development Workshop	Outreach	Peer Mentoring	
	(*n* = 23)	(*n* = 23)	(*n* = 20)	(*n* = 21)	(*n* = 22)	(*n* = 18)	(*n* = 19)	(*n* = 22)	(*n* = 16)	(*n* = 21)	(*n* = 10)*	(*n* = 23)
***Network Science & Interdisciplinarity***												
(1) Learn about important research results in network science	70%	39%	85%	43%	32%	28%	11%	59%	19%	5%	10%	100%
(2) Learn about the theory and methods of network analysis	30%	91%	60%	33%	41%	22%	11%	27%	13%	0%	10%	96%
(3) Develop an understanding of methods of other fields	96%	43%	70%	86%	59%	44%	21%	59%	44%	14%	40%	100%
(4) Apply methods of network analysis to biological data	52%	78%	20%	33%	32%	22%	16%	18%	6%	10%	10%	91%
***Communication***												
(5) Develop skills for oral presentations	96%	35%	80%	95%	14%	11%	89%	45%	25%	29%	20%	100%
(6) Learn to build effective visualizations	87%	30%	0%	33%	27%	11%	26%	36%	6%	10%	0%	91%
(7) Develop written communication skills	87%	22%	15%	33%	32%	17%	26%	9%	0%	0%	0%	91%
(8) Communicate to a diverse audience	96%	43%	50%	81%	27%	17%	74%	45%	19%	48%	40%	96%
***Career Preparation***												
(9) Build teamwork skills	70%	70%	50%	19%	18%	17%	63%	5%	6%	33%	30%	96%
(10) (10) Gain experience mentoring others	17%	22%	15%	5%	0%	6%	47%	5%	6%	29%	50%	78%
(11) (11) Develop leadership skills	22%	26%	10%	0%	5%	0%	47%	9%	0%	33%	50%	65%
(12) (12) Gain exposure to diverse career options	17%	9%	25%	33%	18%	22%	16%	45%	69%	0%	10%	78%

*Note*. Percentages reflect the number of students who endorsed that the objective was met by each component out of the total number of students who reported participating in or endorsed an objective for that component. Percentages are shaded such that darker cells indicate a larger percentage of students reported that objective was promoted by that component.

* Only data from cohort 2 is shown for Peer Mentoring due to differences in survey design.

**Fig 6:**
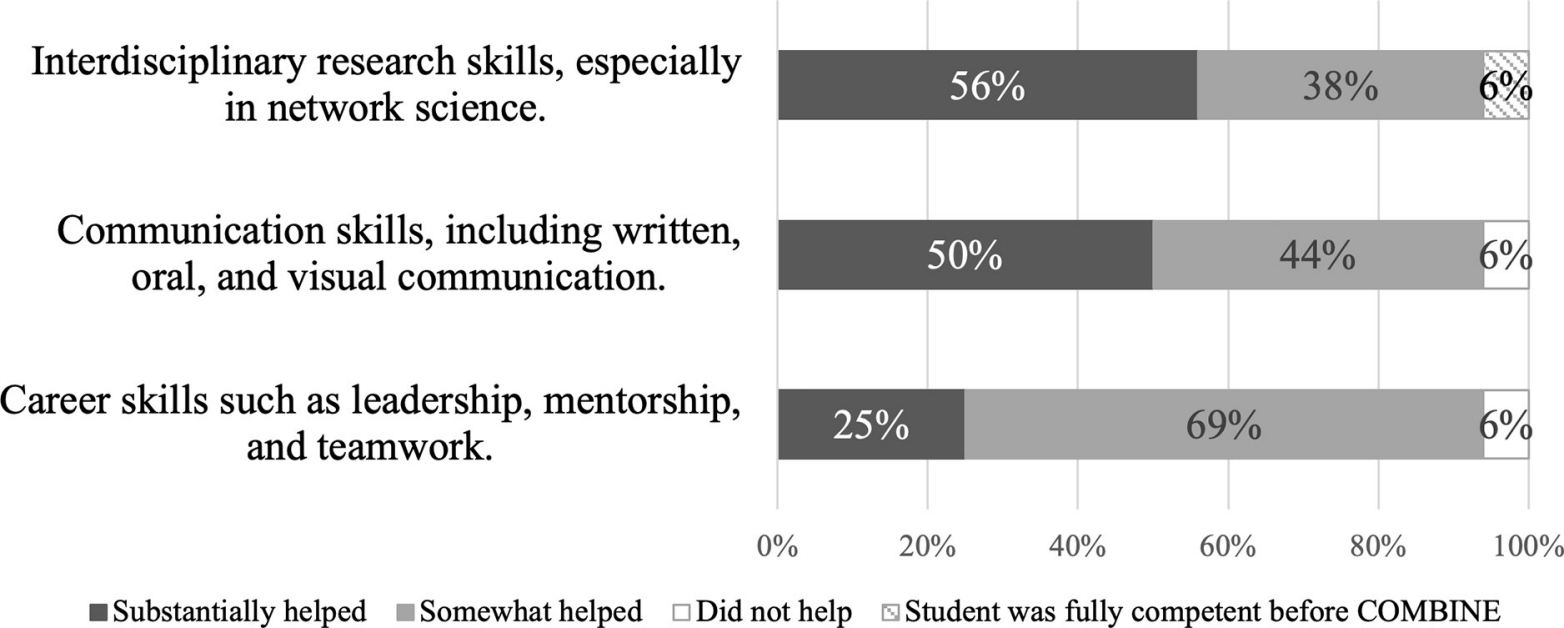
Faculty advisor ratings of how helpful the COMBINE program was to their student in the three program objective areas. *Note*. Percentages were calculated out of the total number of survey responses *(n* = 16). Percentages may sum to greater than 100% due to rounding.

The data on students’ experiences from the perspective of their faculty advisors were drawn from the faculty advisor post-program survey. The quantitative data presented here (Fig 6) were drawn from advisors’ responses to a scale-response question, “Indicate how much the COMBINE program helped your student to develop the following skills,” with the following single response options: “unable to assess,” “student was fully competent before COMBINE,” “COMBINE did not help at all,” “COMBINE helped somewhat,” “COMBINE helped substantially.” No advisors selected “unable to assess” and thus that response option omitted from Fig 6. The qualitative descriptions of the student experience according to their advisors were drawn from two open-ended questions in the post-program survey: “How has your student used what they gained in COMBINE (e.g., how has your student applied the skills/ knowledge/ network that they gained from COMBINE)?” and “List the most important way(s) the COMBINE program benefited your student.”

The open-ended responses from the surveys, interviews, and focus groups were utilized to illustrate the themes in the quantitative results. Two authors who are also members of the evaluation team (KEM and GMA) separately reviewed all of the open-ended responses prior to conducting quantitative analyses, separately identified quotes that represent the data, and then together determined quotes for inclusion. All quotes have been edited for conciseness and clarity; verbatim quotations are available upon request.

## Results

Overall, students reported that the COMBINE program helped them to meet the objectives (Table 6) and found the components to be useful (Fig 5). Likewise, the majority of advisors reported that they thought the program was useful in promoting their students’ skills in each of the three objective areas (Fig 6). For most objectives (9 of 12), over 90% of students felt that the objective was met by at least one program component (Table 6, right-most column). The three remaining objectives were still met by at least one program component for the majority (>65%) of students. Similarly, all but one of the components (Peer Mentoring) were rated as useful by the majority of students (> 60%, Fig 5). Students’ reports generally mirrored the degree to which the components were designed to target each objective (compare the color coding of Tables 3 and 6). On the whole, students’ reports reflect the complementary design of the COMBINE components: the core courses address most objectives, and directly target the Network Science & Interdisciplinarity objectives, while the broadening activities support the Communication and Career Preparation objectives.

### T-shaped portion of the COMBINE model

The vertical leg of the T-shaped model comes from the student’s in-depth training in their home domain. The horizontal leg of the T-shaped model is designed to help students gain familiarity with domains outside their own that are relevant to their research goals. Within COMBINE, the horizontal part of the T is specifically connected to the Network Science & Interdisciplinarity objective 3, “*develop an understanding of methods of other fields*.” The components in which this objective is intended to be directly targeted, the discipline-bridging courses, were rated as contributing to their knowledge of the methods of other fields by 44–59% of students. This may reflect the high level of variability in the courses students selected to fulfill the discipline-bridging course requirement. Although only about half of the students felt the discipline-bridging courses supported the targeted objective, the courses were rated as highly useful (Fig 5). For example, one student from computer science spoke about the discipline-bridging course saying,

…that was actually one of my favorite parts of COMBINE because it was computational neuroscience. I’m trying to be in the middle of [artificial intelligence] and neuroscience–I already completed the classes for my program–and this sort of forced me to take a class that I wouldn’t have otherwise.

This sentiment was echoed in the open-ended survey responses from students’ advisors. To illustrate, one advisor noted that their student applied a “certain scientific way of thinking [and an] interdisciplinary approach to science” because of COMBINE. Another stated that the most important way that COMBINE benefited their student was “the exposure to different research that spanned networks and biological questions.”

### Shield-shaped portion of the COMBINE model

The shield-shaped portion of the COMBINE model (Fig 3) is aimed at providing moderate-depth training in Network Science & Interdisciplinarity (Table 1, objectives 1, 2, and 4). More than 90% of students reported that each of the Network Science & Interdisciplinarity objectives were met by at least one program component (Table 6, right-most column) and 100% of the advisors reported that they felt the COMBINE program helped their student grow at least somewhat in interdisciplinary research skills, particularly in network science (Fig 6). Notably, 100% of students felt that the program supported their growth in objective 1, “*learn about important research results in network science*.” The core coursework, COMBINE seminars, and annual symposium were the components that most students reported contributed to their growth in the Network Science & Interdisciplinarity objectives–all of which were rated as useful by at least 70% of students (Fig 5). To illustrate, one student said, “…without COMBINE… I would have lacked the enrichment in understanding applications of networks beyond the current state of research in my field.” Another student said, “In [COMBINE], I got a lot more fundamental [concepts]–… I wouldn’t call it discipline bridging, but fundamental network science concepts that are consistent across applications.”

Similarly, advisors also described growth in the Network Science & Interdisciplinarity objectives, with one advisor saying…

One of the biggest benefits to [my student] was learning new network biology analysis techniques that enabled [them] to perform more meaningful sequence analyses and visualizations on the dataset that [they] created and analyzed for [their] dissertation. . . . [they] greatly improved [their] interdisciplinary research skills through the COMBINE program.

It is important to note that while most students reported that the program supported their growth in the Network Science & Interdisciplinarity objectives, the specific knowledge gained and/or effort required to learn those skills likely varied depending on students’ domain and/or individual background. Though we lack sufficient sample size to statistically compare students’ reports by discipline, this pattern is suggested by the students’ qualitative responses. One computer science student explained, “There was a lot of bio jargon in my first semester of COMBINE, especially in genetics. Also, not knowing R made the network analysis course more difficult.” A physics student expressed a similar sentiment; they identified the two biggest barriers faced in COMBINE as “My lack of knowledge in network biology especially compared to the computer science students. And my lack of knowledge of specific biology/computer science topics that students were working on.”

### Pi-shaped portion of the COMBINE model

The objectives targeting collaboration, the pi-shaped portion of the model (i.e., Communication and Career Preparation), were also largely met by the program, though more students found the program supported objectives related to Communication compared to Career Preparation.

#### Communication

Students reported that the program largely met all of the communication objectives, with over 90% of students feeling that at least one program component helped them improve their skills in each objective (Table 6, right-most column), and 94% of advisors reported that COMBINE helped their student develop their communication skills at least somewhat (Fig 6). All students felt that the program helped them to “*develop skills for oral presentations*.” These objectives were predominately supported by the data practicum course; at least 87% of students reported the course supported their growth in each of the communication objectives and 100% of students endorsed the course as useful (see [36] for additional information). The seminars, particularly the Research-in-Progress seminar, and the Peer-to-Peer Tutorial (P2P) were components that students also felt contributed to their growth in communication and were rated as useful by a majority of students (90% and 74%, respectively, Fig 5). These quantitative ratings were further illustrated by students’ descriptions of their experiences, as communication was the most common area discussed by students (Box 1), and advisors’ responses to open-ended survey questions. One advisor stated that the biggest benefit their student received from COMBINE was that it “provided a community and gave [my student] feedback from someone other than me on [their] research communication (presentation and writing).” Another said their student “learned graph visualization software in [the Network Analysis course] and used it to make figures for [their] oral presentations and dissertation.”

**Box 1. Students’ descriptions of communication skills gained in COMBINE**

**Communicating with diverse audiences:**

“The interdisciplinary skills that I gained were more than the networks … seeing some different kinds of talks and understanding … how I can present my work to be understandable by different people. I think that the skills that I gained for that are pretty solid.”

“The biggest benefit I got from that program is to think in that interdisciplinary way and how to cast my research in a way that will be accessible to someone outside the field and still excite them. Everyone in the program to a certain degree have started to think that way. Just the fact that people’s attitudes have started to change help achieve that goal of creating more interdisciplinary researchers.”

“The data practicum course was very useful in learning how to communicate concepts from one field to an audience from another.”

“The paper I plan on submitting will probably go to a very interdisciplinary journal so that’s helping me to write in that style and just remembering you’re in the lens of a physicist but I need to portray it to biologists, chemists, so and also in terms of presentations, like the poster presentations.”

**Working in teams or with peers:**

[Working in teams of four in the seminar for presentations] “It was kind of cool to [have] one person from the computer, mathematical, physics and then one person from the bio, neuro side of it. There’s one person who’s more about the algorithms and the network science side and then one person who’s more on the application side of it.”

“[*COMBINE] definitely improved my skills in working with groups just because of the sheer amount of group work*.*”*

“I was just going to say, it depends on if you like your partner but [they] could open up the door to collaborations. If you get to know somebody more and more then you get to know their research, you know?”

“[I learned] how to work/collaborate with an interdisciplinary team (through the paper presentation).”

#### Career preparation

Though still a majority, fewer students reported that the program supported their growth towards the Career Preparation objectives compared to the other two objective areas. Only 65% of students reported that the program helped them to “*develop leadership skills*.” These results are likely somewhat due to the flexibility of the program. The opportunities for students to develop leadership skills had to be, in part, selected by the students (e.g., taking on a leadership role on the student committee) and the value of these opportunities varied based on students’ needs. For example, a student who took advantage of leadership opportunities and found them to be valuable, said “people that were part of the committee had extra opportunities to lead programs, to take charge of certain program elements, build their resume, and give them other things to talk about during interviews.” Another student saw the opportunities were available but preferred to focus on other aspects of the program, saying “I do think I learned some leadership skills but I would have liked to be able to use those opportunities to do more interdisciplinary stuff.” For the only component designed to promote leadership directly (P2P), less than half (47%) of students reported that it helped them meet this objective. Despite this, the component was still rated as useful by 74% of students (Fig 5), possibly due to a perceived contribution to other objectives, like communication and teamwork.

For mentoring skills, 78% of students felt the program as a whole supported their growth, and half of students (50%) reported receiving support from the component that was intended to directly target mentoring skills, Peer Mentoring. Interestingly, Peer Mentoring was also rated as useful by fewer students than any other component. No students rated Peer Mentoring as very useful and only 30% of students rated it as somewhat useful. Taken together, these results suggest that many students received support from components that indirectly or were not anticipated to target mentoring skills (e.g., P2P, Outreach) and that the direct, intended approach to promoting mentoring skills (i.e., Peer Mentoring) was largely not seen as useful by students in the first two cohorts.

Additional insight into the discrepancy between the number of students who felt Peer Mentoring supported their grown in mentorship skills (50%) and those who found it useful (30%) comes from the students’ qualitative responses. Students generally understood that the program was aiming to enhance their mentorship skills, yet did not find the formal Peer Mentoring to be particularly beneficial to their own development in the way it was implemented for the first two cohorts. For example, one student explained that Peer Mentoring “could be implemented better or not be required… there are so many places for us to get mentoring. We do that unofficially among each other and among people at our home departments and programs.” Based on students’ feedback, the Peer Mentoring component was adapted for subsequent cohorts, for which data are not yet available.

It is notable that the three objectives that were reported as least met by the program (i.e., mentorship, leadership, and exposure to diverse career options) were also the only objectives designed to be directly targeted by a single program component. As such, these results suggest an area to improve the program design. It may be that objectives need to be directly targeted by multiple components, both structured and unstructured, in order to be fully met.

This is the one objective area in which the advisor and student responses diverged slightly, though this likely reflects methodological differences in how and when students and advisors were surveyed. Advisors were surveyed at least six months after students completed the program and were asked how beneficial the program was to their students career preparation overall. The advisors largely reported that COMBINE was beneficial to their students’ career preparation, with 94% saying the program helped their student at least somewhat (Fig 6). Several also described career preparation benefits as being the most important way their student benefited from the program. For example, one advisor described how their student was already applying skills they learned in COMBINE in their career saying “They are currently a data analyst at the National Institutes of Health working with large open datasets [and] much of the computational aspects of the program are useful for this.” Meanwhile, students were surveyed immediately upon completing the program and were asked whether each specific component improved their skills in each career preparation objective individually (e.g., mentoring skills). As such, this slight discrepancy may simply reflect a difference in perspectives such that advisors were taking a broader view of their students’ overall preparedness to enter the workforce and students were more narrowly focused on a small set of specific skills. Anecdotally, alumni who were interviewed described the career benefits they derived from the COMBINE program.

### Integrating the three models

One of the strengths of the COMBINE model is the integration of the T-shaped, pi-shaped, and shield-shaped models to promote both students’ network science skills and their collaboration skills. This integration is illustrated in Table 6 with the balance of network science objectives, which most students reported were addressed by the COMBINE courses, and collaboration skills, which students reported were largely addressed by the core courses and broadening activities. The value of this integrated approach can be seen not only through the student reports described above, but also through the students’ research products in network science which include 28 published or accepted peer-reviewed articles, 27 conference presentations, and alumni feedback. After graduation, a student described how they used both the knowledge they gained in network science, as well as communication and mentorship skills, to supervise a project in their job, saying…

I am supervising someone through a network neuroscience project. He’s a premed student who didn’t have any experience doing this type of thing. So it’s been helpful to be able to supervise him through it. I definitely utilized skills I learned in COMBINE for that.

The same student elaborated on the value of the collaboration skills they learned saying…

In my graduate lab, everybody knew all the jargon and things that we were talking about. But with COMBINE, I got a lot of practice talking with people from different disciplines. And now I’m in really big projects. We have physicists and computational biologists and cognitive neuroscientists and geneticists that we collaborate with, and we need to be able to speak the same language at a certain degree. I use those skills every day.

Another student described the value of collaborating with other domains to work on network science problems in their courses, saying…

You can have people come up with other applications of the same methods… How would you potentially apply it to another field? … If you’re working with someone who does ecological networks and we’re doing a neuroscience paper how can we use this to analyze ecological networks?… It was cool to have one person from computer, mathematics, and physics and then one person from the bio, neuro side of it. There’s one person who’s more about the algorithms and the network science side and then one person who’s more on the application side of it.

Similarly, several advisors described the most important benefits to students as a combination of the objective areas. To illustrate, one advisor said “I expect the most important benefit to come from the broader science perspective [they] obtained [is that it] will benefit [them] in [their] career as [they] start a postdoc or industry position,” capturing both the shield-shaped and pi-shaped portions of the COMBINE model. Another advisor said the most important benefit their student experienced was a “deeper understanding of network science, dynamical systems and interdisciplinary research. [Also], improved communication skills, especially in communicating with others in different disciplines,” a sentiment that captures the t-shaped, shield-shaped, and pi-shaped aspects of the COMBINE model.

## Lessons learned

The COMBINE program provides an integrated approach to training graduate researchers to work at any point along the spectrum of interdisciplinarity (Fig 1). The program expands their expertise in knowledge, data, and methods across disciplines, as well as improves their collaboration skills, both of which are essential aptitudes for addressing complex problems [14, 15]. Students reported feeling that the program supported their growth in network science, interdisciplinarity, communication, and career preparation. It must be noted that both the program description presented here and the representations of students’ experiences, are limited to things that were formalized, operationalized, and measured. While we are unable to further demonstrate it empirically, these formalized pieces coalesce together to create a lively scholarly environment with an engaged scientific community. Here, we describe some of the lessons learned and conclusions from the COMBINE program.

### A complement rather than replacement to traditional graduate programs

It may be tempting to conclude that a new doctoral program is the ultimate goal of graduate education in network science. Yet one of the strengths of COMBINE is its structure as an additional program that students from many different disciplines can complete en-route to their doctorate. The interaction of students and faculty from different academic silos, all working on issues under the network science umbrella, allows for a truly integrated interdisciplinary experience. It is not the goal of COMBINE to replace the specific expertise and academic identity found within student’s home departments, but rather to build upon them and create a framework and participatory culture in which researchers from different silos can work together [15, 31]. In order to successfully do this in network science, and in interdisciplinary research broadly, researchers must share some baseline expertise in adjacent fields as well as have the collaboration skills to work together effectively [14, 15]. By complementing rather than replacing students’ training in their home disciplines, COMBINE benefitted from students’ in-depth knowledge and that of their advisors. COMBINE only had to supplement that knowledge with moderate expertise in network science and was able to do so, in part, by allowing the students to share their expertise in adjacent disciplines with one another. This peer education not only facilitated students’ growth in other areas, but also gave students practice in communicating and working with others from different backgrounds. The assumption that students are gaining specific expertise in their home program allows COMBINE space and attention to focus on integrated interdisciplinary work.

The complementary-to-home-domain approach, which includes opportunities for learning both in and outside the classroom, also provides students with training on how to communicate and collaborate specifically with an audience of people working in network science applications. This breaks down several of the barriers to interdisciplinary work by creating a shared language, developing competency to understand the questions asked and methods used by other fields, and providing insight into the cultural norms of other disciplines [19, 34, 35].

However, it is important to note that there are several challenges associated with the complementary approach taken by COMBINE. At times the training or requirements may be redundant depending on the degree of overlap between what a student gets from COMBINE and from their home program. For example, if a student’s home program already includes the opportunity to mentor undergraduate students, doing so again to satisfy the COMBINE outreach requirement may not provide an additional benefit. The COMBINE program attempted to mitigate redundancy by offering flexibility in the program requirements, an approach that has its own challenges discussed in detail later in this section.

### Establishing and maintaining a cohesive and diverse student body

In order for students to be able to work at any level of integration in the spectrum of interdisciplinarity, they must be able to productively work with people from other disciplines [15, 19]. COMBINE provides students with the opportunity to practice this by creating a cohesive community of students from different discipline domains. This was successfully done by intentionally recruiting students from specific programs, establishing quotas for enrollment from each domain, and ensuring that small groups and collaborations in courses and broadening activities were made up of groups of students from differing backgrounds.

Yet diversity extends beyond representation from the various disciplines working in network science. High-quality (graduate) education requires building and supporting a demographically diverse community of students and faculty [1, 32, 40, 41]. This is achieved within COMBINE only to the extent possible based on the existing pool of students and faculty that are already part of relevant doctoral programs. There continues to be a lack of diversity in STEM [18, 42], which is thus reflected in the COMBINE population to some degree.

While this can present some limitations, the complementary nature of a program such as COMBINE could be leveraged to promote and support diversity. The program’s community-building events and peer mentoring initiatives could be spaces for discussion about diversity, equity, and inclusion. Simultaneously, by serving as hub across many programs within an institution, COMBINE could facilitate affinity-based groups or mentoring to support students who might otherwise be isolated.

### Providing consistent, valuable training to students from different disciplines

One of the biggest challenges in COMBINE has been maintaining a consistent standard of knowledge and training when each student needs a different level and/or type of training to reach that standard. Specifically, in network science, a major challenge has been providing consistent training without students having at least some degree of shared background knowledge (e.g., basic familiarity with statistics and computer programming).

COMBINE has attempted to address this challenge in several ways. First, the small groups for network analysis assignments are made up of students from different disciplines, allowing them to benefit from the expertise of one another. Second, COMBINE requires that those students without prior computational training fulfill their 3-credit discipline-bridging requirement with a computation-based course. Finally, the program offers many informal and less-structured opportunities for students to seek out additional support based on their own needs (e.g., broadening activities such as P2P).

Even still, students experienced challenges, particularly in the network analysis course, due to the variability in student’s expertise (e.g., first time using R, unfamiliar with biological applications). These challenges could be due to the fact that the computation-based discipline-bridging course requirement does not ensure that all students are learning the same programming platforms, as COMBINE cannot dictate the content of the elective courses and the program aims to maintain flexibility in program requirements. This challenge may suggest that training in related fields should be more structured and explicitly addressed rather than through electives and informal and unstructured learning opportunities.

### Program flexibility and student choices

One of the hallmarks of the COMBINE program is a certain degree of flexibility in the program requirements. This flexibility allows students to create their own experience which both promotes engagement through participatory learning and allows students to tailor the program to meet their own specific needs [15]. Yet this flexibility means that the skills targeted and the degree to which students receive a benefit from the program vary depending on students’ choices, making it difficult to establish a consistent standard of training and competency goals.

In COMBINE, this manifests primarily in three ways. First, the value of the skills gained appears to vary depending on students’ backgrounds and what is provided in their home program. Second, the type of skills that students gain from different components varies based on both the activities (e.g., mentoring undergraduates vs giving an external COMBINE presentation to satisfy the outreach requirement) and their role (e.g., leader vs attendee) that students select. Finally, the benefits yielded from COMBINE depend in part on the degree to which students capitalize on the opportunities provided beyond the basic requirements. For example, a student who does a COMBINE outreach presentation to an outside department would experience the greatest benefit if they then take advantage of the new connections with faculty by seeking out collaborations or connecting through professional networks.

The challenges associated with providing consistent training to students from diverse backgrounds demonstrate that there are strengths and weaknesses associated with both structured and flexible aspects of the program. Highly-structured, objective-dense program components (e.g., the COMBINE core courses) can be very successful at achieving the intended goals, though some shared foundational knowledge might be required. On the other hand, flexible or objective-sparse aspects of the program (e.g., the COMBINE Annual Symposium, informal social networking), can build community and be valuable for students who need additional support in specific areas, but the consistency of student gains may be limited. COMBINE’s utilization of both formats is one approach to overcoming these challenges.

## Conclusion

The COMBINE program represents an innovative model of interdisciplinary graduate education in network science, with a particular focus on applications to biological systems. COMBINE’s integrated model of interdisciplinary education builds and expands upon existing educational models by providing training in both network science and collaboration. The combination of these two approaches, in addition to the training provided by students’ home programs, is an example of the age-old adage “*the whole is greater than the sum of its parts*.” Standing alone, none of the COMBINE components would yield comparable results, nor would a student accessing each component separately from different organizations experience the same benefits. COMBINE students are trained in network science content with peers from different domains, taught directly how to communicate with those peers and their broader networks, and given professional development all within the context of network science. This not only provides gains in each of those specified areas, but allows for students to truly develop interdisciplinary relationships and have hands-on practice with interdisciplinary collaboration in network science.

This effort is not without substantial challenges and considerations, like those described above. COMBINE requires substantial funding and administrative support in order to provide all program components in a way that fosters a diverse, engaged community. The program must continually strive to maintain a rigorous standard of training while remaining flexible to accommodate students from different backgrounds. Finally, the success and longevity of the program depend on ongoing support, both material and otherwise, from the university faculty and administration.

Even with these considerations in mind, COMBINE offers a new model of interdisciplinary education that could be implemented at other universities and/or in other fields. The program could be replicated for any interdisciplinary field by simply replacing the core courses and seminar with courses in the appropriate field and providing the remaining components to the appropriate audience. As such, the COMBINE model represents an exciting innovation in the field of interdisciplinary graduate education.
